# Tracking *Bacillus anthracis*: The Legacy of MALDI-TOF Biomarkers in Scientific Literature, a Review

**DOI:** 10.1021/acsomega.5c08472

**Published:** 2025-12-18

**Authors:** Jacqueline Roberta Salgado, Monique Cardozo, Samir F. A. Cavalcante, Adriana Marcos Vivoni, Ian Gardel Silva, Valdir F da Veiga-Junior

**Affiliations:** † 28098 Military Institute of Engineering (IME), Gen. Tiburcio Square, 80, Urca, Rio de Janeiro 22290-270, Brazil; ‡ Institute of Chemical, 589908 Biological, Radiological and Nuclear Defense (IDQBRN), Av. das Américas, 28,705, Guaratiba, Rio de Janeiro 23020-470, Brazil; § National Reference Laboratory for Anthrax, 196605 Oswaldo Cruz Institute IOC), Av. Brasil, 4365, Manguinhos, Rio de Janeiro 21040-900, Brazil

## Abstract

*Bacillus anthracis* is a Gram-positive, spore-forming bacterium and the etiological agent of anthrax, a re-emerging, septicemic, hemorrhagic, and often lethal disease that affects humans as well as domestic and wild ruminants. Owing to its high lethality, spore stability, and ease of dissemination, this pathogen is recognized as a major biological-warfare agent and is classified among the Category A bioterrorism threats. Rapid and unequivocal identification is essential; however, it is hindered by the extensive similarity between *B. anthracis* and closely related species, particularly *Bacillus cereus*. Matrix-assisted laser desorption/ionization time-of-flight mass spectrometry (MALDI-TOF MS) has emerged as an increasingly adopted tool in diagnostic laboratories due to its cost-effectiveness, speed, and reliability in microorganism identification (Infographic 1). This comprehensive review compiles known *B. anthracis* biomarkers and evaluates the methodologies employed across scientific studies via systematic bibliographic analysis. We identified 87 *B. anthracis* biomarkers reported in the literature and classified them according to their specificity relative to those of other members of the *B. cereus* group. Moreover, we highlight gaps in methodological standardization and biomarker validation, emphasizing the need to develop robust protocols capable of ensuring the accurate detection of *B. anthracis* in biological defense contexts and surveillance. Accordingly, we propose a standardized analytical workflow aimed at improving diagnostic reliability, with recommended tools and best practices for enhancing the application of mass spectrometry in the specific identification of *B. anthracis.*

## Graphical abstract

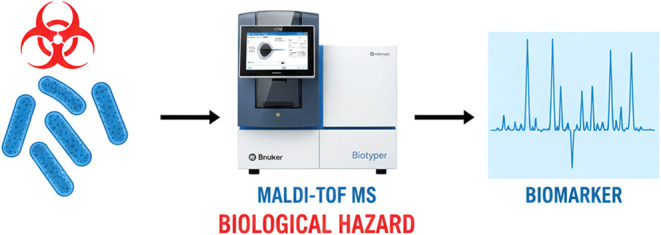

## 1 Introduction

Matrix-assisted laser desorption/ionization time-of-flight mass spectrometry (MALDI-TOF MS) has enabled faster and more reliable analysis and identification of microorganisms, offering the advantages of a general-purpose sample-preparation platform applicable to a wide range of taxa, including bacteria, fungi, and yeasts, while also reducing operational costs.^1–4^ As a diagnostic tool, MALDI-TOF MS facilitates the discrimination of microbial species from both genetic and proteomic perspectives; however, distinguishing closely related species remains a substantial challenge.

*Bacillus anthracis* is a Gram-positive, rod-shaped, spore-forming bacterium within the broad *Bacillus* group that encompasses several genetically related species. It is the causative agent of anthrax, a globally distributed enzootic and zoonotic disease that is septicemic, hemorrhagic, and frequently fatal, primarily affecting wild and domestic herbivores across most countries in Asia and Africa, as well as parts of Europe and the Americas. The species exhibits high virulence, and its spores are remarkably resilient under adverse environmental conditions, remaining viable in soil for many years. Notably, *B. anthracis* spores have been used as biological weapons in powder formulations, as demonstrated by bioterrorism-related letter attacks in the United States in 2001.^5,6^

This dual relevance necessitates rapid and accurate identification methods for clinical, epidemiological, and biosafety purposes. Conventional microbiological and molecular approaches face limitations due to the extensive phenotypic and genetic similarity between *B. anthracis* and other members of the *B. cereus* group. Although MALDI-TOF MS provides advantages in terms of speed, cost, and diagnostic reliability, its application to high-consequence pathogens still requires rigorous methodological standardization and robust validation.^7^

Given the challenges arising from the limited diversity of MALDI-TOF MS spectral libraries for bioterrorism-related microorganisms with high genetic and proteomic similarity between *B. anthracis* and *B. cereus*, this review presents a systematic analysis of current methodologies. We further propose a standardized analytical protocol and identify *B. anthracis*-specific biomarkers to support accurate detection in diagnostic and biodefense settings.

## 2 Biological-Warfare Characteristics and Identification Challenges

### 2.1 *Bacillus anthracis* as Biological Threat

Bioterrorism is a form of terrorism in which biological agents are employed as weapons against humans, animals, or crops, with the primary objective of intimidating civilian populations and authorities as well as advancing the political, religious, or ideological aims of the perpetrators.^8^ *Bacillus anthracis* possesses several characteristics that make it particularly suitable for biological-warfare applications: high lethality following inhalational exposure (mortality >80% in the absence of treatment), ease of production and dissemination, remarkable environmental stability attributable to its spore-forming capacity, and an ideal particle size (1–5 μm) for deep deposition within the respiratory tract.^9^

Contamination may result from a single exposure event, with cases potentially detected across different locations and at varying times due to the incubation period of the disease. Disease progression may also differ among exposed individuals depending on factors, such as overall health status and pre-existing immunity. Distinguishing a deliberate biological attack from naturally occurring infectious diseases can therefore be challenging, particularly in unannounced events.^10–12^

The 2001 anthrax attacks in the United States illustrated the profound socioeconomic consequences of bioterrorism, resulting in 22 confirmed cases (11 inhalational and 11 cutaneous), five fatalities, prophylactic treatment of more than 30,000 individuals, and direct economic costs exceeding US$6 billion. These events underscored critical gaps in rapid diagnostic capability and reinforced the urgent need for improved methods of early detection and identification.^13,14^

### 2.2 Identification Challenges

*Bacillus anthracis* belongs to the phylum Firmicutes, family Bacillaceae, genus *Bacillus*, and is part of the *B. cereus* group (also referred to as the large *Bacillus* group or the *B. cereus* *sensu lato* group). Microorganisms within this genus are ubiquitous and can be isolated from soil, seawater, freshwater, and various food products. These bacteria produce spores—resistant, multilayered structures capable of remaining dormant for decades and, under ideal conditions, even millions of years.^15–17^ The principal pathogenic characteristics of this group are attributed to toxins that are typically encoded on plasmids.^18–20^

*B. anthracis* harbors two plasmids that are essential to its virulence: pXO1 (182 kb), which encodes toxin synthesis, and pXO2 (96 kb), which encodes the poly-γ-d-glutamic acid capsule. Both plasmids may be transferred to related species under certain circumstances, particularly within the large *Bacillus* group.^21,22^ Among *B. anthracis* strains, noncapsulated variants (pXO1^+^/pXO2^–^) have been identified in environmental samples and sometimes co-occur with virulent strains.^23^ The presence of both plasmids is considered crucial for the identification of pathogenic *B. anthracis*, and the loss of either plasmid results in attenuated virulence, while the loss of both eliminates it entirely.^24–27^

*B. anthracis* has a worldwide distribution, with regions classified as hyperendemic, endemic, or sporadic. Global estimates indicate that between 2000 and 20,000 human cases occur annually. However, according to the World Health Organization, the true incidence may be up to ten times higher, as reporting is not mandatory in many countries and official data are absent or incomplete in others.^28–31^

Within the *B. cereus* *sensu lato* group, plasmid transfer (or partial plasmid transfer) may occur, giving rise to *B. cereus* strains that are “anthracis-like,” harboring plasmids highly similar to those of *B. anthracis* and capable of causing anthrax-like disease. Of particular importance is *B. cereus* biovar *anthracis* (Bcbva), which contains pXO1 and pXO2 and produces a disease in animals that is clinically indistinguishable from classical anthrax.^21,33,34^

Since 2004, atypical cases of *B. cereus* strains causing inhalational anthrax-like disease in humans and other mammals have been reported. These strains harbor pXO1-like plasmids (pBCXO1) exhibiting approximately 99.6% nucleotide identity to the *B. anthracis* pXO1 plasmid.^35^ In Cameroon (CA) and Côte d’Ivoire (CI), Bcbva strains have been identified in chimpanzees, gorillas, monkeys, elephants, and various livestock, causing large-scale anthrax-like outbreaks in West Africa (Table 1).^21,22,34,36–42,44,45,47–51^

**Table 1:** Description of Atypical Strains and Bcbva, Including Their Location and Source of Origin, Virulence Plasmid, and Disease

organism	country of origin	host	virulence plasmids	references
*B. cereus* G2941	Louisiana, USA	Human; blood/sputum	pBCXO1	^21^
			pBC210	
*B. cereus* 03BB87	Texas, USA	Human; blood	pBCXO1	^36^
			pBC210	
*B. cereus* 03BB102	Texas, USA	Human; blood	pBCXO1	^36,38,39^
			pBC210	
*B. cereus* Elc2	Texas, USA	Human; blood	pBCXO1	^37,38^
*B. cereus* Fl2013	Florida, USA	Human; skin lesion swab	pBCXO1	^40^
*B. cereus* La2007	Louisiana, USA	Human; unknown sample	pBCXO1	^38^
			pBC210	
*B. cereus* JF3964	Koza, Cameroon	Bovine	pBCXO1	^39^
			pBCXO2	
Bcbva Ca	Dja Reserve, Cameroon	Chimpanzee and gorilla	pBCXO1	^22,44,45^
			pBCXO2	
Bcbva CI	TaT National Park Cote’lvoire	Chimpanzee	pBCXO1	^22,44,45^
			pBCXO2	
*B. cereus* G9898	Louisiana, USA	Human; blood/sputum	pBCXO1	^50^
			pBC210	
*B. cereus* Bk-Ak	China	Kangaroo	pBCXO1	^51^
			pBCXO2	

The U.S. Department of Health and Human Services has added Bcbva to its list of select agents for potential use in bioterrorism, owing to its strong similarities to *Bacillus anthracis*.^50^

Difficulties in identifying *B. anthracis* arise primarily from its high phenotypic and genetic similarity to *B. cereus* and other closely related species. In environmental samples, *B. anthracis* is typically present as spores, whereas in clinical specimens, it appears as vegetative cells. Consequently, the strategies required for its detection differ according to the type of sample being analyzed.^51–55^

Identification of *B. anthracis* is commonly based on morphological characteristics; biochemical tests such as motility, hemolysis, and lecitinase activity; γ phage susceptibility; penicillin sensitivity; and molecular methods, including PCR targeting chromosomal and plasmid genes. Despite their utility, these approaches have several limitations. The turnaround time is relatively long, typically requiring 18–48 h for definitive identification. Phenotypic variability further complicates diagnosis as some strains exhibit atypical characteristics. In addition, the methods involve considerable technical complexity, demanding specialized expertise, and the use of high-level biosafety facilities. They are also costly, relying on expensive reagents and equipment. Finally, concerns regarding reliability persist, given the potential for false-positive or false-negative results arising from strain-to-strain variation. These constraints highlight the need for cost-effective, high-throughput, and reliable methodologies for diagnosing infectious diseases, with particular emphasis on the early detection of *B. anthracis* in humans and animals.^56–60^

Other mass spectrometry-based methods, such as gas chromatography–mass spectrometry (GC–MS) and matrix-assisted laser desorption/ionization mass spectrometry (MALDI-MS), have also been applied.^61^ While several studies demonstrate that MALDI-MS can distinguish *B. anthracis*, the high degree of similarity between the spectral fingerprints of *B. cereus* and *B. anthracis* has been reported as a significant obstacle.^62^

## 3 MALDI-TOF MS Biomarker Analysis

This technique involves three main stages: ionization, analysis, and detection. Initially, the microbial sample is mixed with an organic compound known as a matrix and cocrystallized on a metal plate. A pulsed laser beam is then directed at these crystals. The matrix absorbs the laser energy and transfers it to the analyte molecules—primarily proteins—facilitating their desorption from the plate and ionization, typically through protonation ([M + H]^+^).^1^ The resulting ions are subsequently accelerated by an electric field into a vacuum tube, the time-of-flight (TOF) analyzer, where they travel toward a detector. The time required for each ion to traverse this distance is measured with high precision: ions of lower mass reach the detector more quickly, whereas higher-mass ions require longer transit times. From these measurements, the mass-to-charge (*m*/*z*) ratio of each ion is calculated.^1,2^

For microorganisms, MALDI-TOF MS is typically employed to generate a proteomic fingerprint largely composed of ribosomal proteins and other conserved, abundant cellular proteins characteristic of each species. The resulting mass spectrum is compared against a reference database containing spectra from previously characterized organisms. A high degree of concordance between the sample spectrum and reference entries enables rapid and accurate species-level identification and, in some cases, subspecies-level resolution. For *B. anthracis*, this technique has been widely applied to the comprehensive characterization of bacterial proteomes, including vegetative cells with varying plasmid content, mature spores, and germinating spores.^1,4^

Biomarkers detectable by MALDI-TOF MS may originate not only from proteins but also from other biochemical classes such as lipids and small organic molecules. The identification of unique biomarkers is feasible due to the rich taxonomic information contained in these molecules and offers promising avenues for detecting a broad spectrum of biological agents. Another potential biomarker is dipicolinic acid (DPA), found in very high concentrations in bacterial spores when complexed with calcium, though it is not specific to *B. anthracis*.^63,64^

Mass spectrometry thus represents a powerful tool for the discovery and identification of protein markers.^65^ In the case of *B. anthracis*, this approach has been extensively used for proteomic characterization, particularly of vegetative cells with different plasmid compositions.^66–70^

## 4 Results and Discussion

### 4.1 Biomarker Analysis

A total of 87 biomarkers associated with the identification of *Bacillus anthracis* were reported across 16 studies published between 1996 and 2025. These biomarkers encompass multiple molecular classes, including ribosomal proteins, small acid-soluble proteins (SASPs), and other cellular components, with mass-to-charge (*m*/*z*) values ranging from 2385 to 7744 (Table 2).

**Table 2:** *Bacillus anthracis*’ Biomarkers

biomarker(*m*/*z*)	proposed chemical attribution	typical *B. anthracis* strains	analytical specificity vs *B. cereus* group	indicator type	references
2385	Low molecular weight peptide	Sterne, Vollum, Zimbabwe	Low	General profile	^71^
2445	Not assigned	Vollum	Low	Present in *B. cereus* group	^72^
2525	Low molecular weight peptide	Vollum ATCC 14578, Sterne, Delta	Low	General profile	^71^
2603	Not assigned	Sterne	Potential	Present only in *B. anthracis*	^73^
2760	Low molecular weight peptide	Vollum ATCC 14578, Sterne, Delta	Low	Close mass variations	^72^
2789	Vegetative cell protein	Sterne, Vollum, Zimbabwe	Low	General profile	^71^
2792	Low molecular weight peptide	Vollum ATCC 14578, Sterne, Delta	Low	General profile	^76^
2850/2853	Vegetative cell protein	Sterne, Vollum, Zimbabwe, BA10, ATCC14186	Low	Present in *B. cereus* group	^71,74^
2994	Not assigned	Sterne	Potential	Present only in *B. anthracis*	^73^
3068	Vegetative cell protein	Sterne, Vollum	Low	General profile	^75^
3077	Low molecular weight peptide	Vollum ATCC 14578, Sterne, Delta	Low	Close mass variations	^76^
3203	Not assigned	Sterne	Potential	Present only in *B. anthracis*	^73^
3214	Vegetative cell protein	Sterne, Vollum	Low	General profile	^75^
3261/3262	Low molecular weight peptide	Vollum ATCC 14578, Sterne, Delta	Low	Close mass variations	^76^
3339	Spore protein	Sterne, Vollum	Potential	Spore profile	^76^
3353	Not assigned	Sterne	Potential	Present only in *B. anthracis*	^73^
3396	Not assigned	Sterne	Potential	Present only in *B. anthracis*	^6,77^
3450	Not assigned	Sterne	Potential	Present only in *B. anthracis*	^73^
3590/3591/3592	Vegetative cell protein	Vollum ATCC 14578, Sterne, Delta	Potential	Strain discrimination	^76^
3682/3683	Vegetative cell protein	Sterne, Vollum	Low	Present in *B. cereus* group	^6,75^
3991	Low molecular weight peptide	Sterne, Vollum, Zimbabwe	Low	Spore profile	^71^
4063	Vegetative cell protein	Vollum ATCC 14578, Sterne, Delta	Potential	Strain discrimination	^76^
4180	Not assigned	Sterne	Low	General profile	^79^
4248	Not assigned	Sterne	Potential	Present only in *B. anthracis*	^73^
4301	Vegetative cell protein	Vollum ATCC 14578, Sterne, Delta	Low	Close mass variations	^76^
4304	Vegetative cell protein	V770-NP1R	Low	Close mass variations	^76^
4310	Vegetative cell protein	Vollum ATCC 14578, Sterne, Delta	Low	General profile	^76^
4313	Spore protein	Sterne, Vollum, Zimbabwe	Potential	Spore profile. Present only in *B. anthracis*	^71^
4327	Vegetative cell protein	Vollum ATCC 14578, Sterne, Delta	Low	General profile	^76^
4333/4334	Vegetative cell protein	Sterne, Vollum	Low	Present in *B. cereus* group	^75,80^
4440	Not assigned	Vollum	Low	Present in *B. cereus* group	^72^
4505	Vegetative cell protein	Sterne, Vollum, Zimbabwe	Potential	Strain discrimination	^71^
4606	Vegetative cell protein	Sterne, Vollum	Low	Present in *B. cereus* group	^75^
4705	Vegetative cell protein	Sterne, Vollum	Low	Present in *B. cereus* group	^80^
4833	Vegetative cell protein	Sterne, Vollum	Low	Present in *B. cereus* group	^80^
4871	Vegetative cell protein	Sterne	Low	General profile	^77^
4933	Vegetative cell protein	Sterne, Vollum	Low	Present in *B. cereus* group	^80^
5096	Not assigned	Sterne	Low	General profile	^79^
5108	Vegetative cell protein	Sterne, Vollum	Low	Present in *B. cereus* group	^80^
5171	Vegetative cell protein	Sterne, Vollum	Low	Present in *B. cereus* group	^75,81^
5382	Not assigned	Sterne	Low	General profile	^79^
5413	Vegetative cell protein	Sterne, Vollum	High	Main marker for vegetative cells	^73,75^
5476	Not assigned	Sterne	Potential	Present only in *B. anthracis*	^6^
5886	Vegetative cell protein	Sterne, Vollum	Low	Present in *B. cereus* group	^75^
5887	Vegetative cell protein	Sterne, Vollum	Low	Present in *B. cereus* group	^75,81^
6193	Not assigned	Sterne	Low	General profile	^79^
6357/6358/6365	Intermediate molecular weight peptide	Vollum ATCC 14578, Sterne, Delta	Low	Close mass variations	^76^
6610.6	Not assigned	Sterne	Potential	Present only in *B. anthracis*	^6^
6657/6662/6667/6668	Spore protein	Vollum ATCC 14578, Sterne, Delta	Low	Close mass variations	^76^
6675	Spore protein	Sterne, Texas, Vollum	Low	Present in *B. cereus* group	^73,82^
6678/6679/6680	Spore protein	Broad spectrum of *B. cereus* group	Low	Present in *B. cereus* group	^79,83^
6684	Spore protein	Sterne, Vnr-1	Potential	Close mass variations	^84^
6695	Spore protein	Sterne, Zimbabwe	Low	Present in *B. cereus* group	^32,43^
6698	Spore protein	Sterne, Vnr-1	Potential	Present only in *B. anthracis*	^84^
6711	Spore protein	Sterne, Zimbabwe	Low	Present in *B. cereus* group	^32,43^
6712	Spore protein	Sterne, Vollum	Low	Present in *B. cereus* group	^80^
6753	Spore protein	Sterne, Vnr-1	Potential	Present only in *B. anthracis*	^84^
6811/6816/6820/6825	Spore protein	Vollum ATCC 14578, Sterne, Delta	Low	Close mass variations	^73^
6834/6835	Spore protein	Broad spectrum of *B. cereus* group	Low	Present in *B. cereus* group	^80,83^
6836/6837	Spore protein	Sterne, VNR-1, ANR-1, Vollum, Zimbabwe	Low	Close mass variations	^43^
6840	Spore protein	Sterne, Vnr-1	Potential	Close mass variations	^84^
6885	Not assigned	Sterne	Low	General profile	^79^
7065	Spore protein	Vollum ATCC 14578, Sterne, Delta	Low	Close mass variations	^76^
7080/7081	Spore protein	Sterne, Vollum, VNR-1, Zimbabwe	Low	Present in *B. cereus* group	^32,76,80,83^
7331	High molecular weight peptide	Sterne, Vollum	Low	Present in *B. cereus* group	^80^
7365	High molecular weight peptide	Sterne	Potential	Present only in *B. anthracis*	^6^
7367/7368	High molecular weight peptide	Sterne, Vollum	Low	Present in *B. cereus* group	^75,81^
7744	High molecular weight peptide	Vollum ATCC 14578, Sterne, Delta	Potential	Present only in *B. anthracis*	^76^

In 1996, Krishnamurthy and collaborators conducted the first study applying MALDI-TOF MS to the identification of *Bacillus anthracis*. Using irradiation for pathogen inactivation, they described mass peaks at *m*/*z* 6679, 6835, and 7082 in spectra from the *B. anthracis* Zimbabwe strain. In 1999, Hathout and colleagues confirmed the peaks at *m*/*z* 6679 and 6835 in spores of the *B. anthracis* Sterne strain.^78^ In 2000, Ryzhov, Hathout, and Fenselau identified only *m*/*z* 6678 as being specific to *B. anthracis*.

In 2001, Elhanany and collaborators investigated biomarkers of *B. anthracis* spores and other members of the *B. cereus* group (*B. cereus*, *B. thuringiensis*, and *B. mycoides*), as well as more distantly related *Bacillus* species such as *B. subtilis* and *B. licheniformis*. However, the putative biomarkers detected in the 4–10 kDa range exhibited only moderate mass accuracy in positive linear mode.

In 2003, Hathout et al. revisited the peaks at *m*/*z* 6679, 6835, and 7082 and demonstrated that they corresponded to small acid-soluble proteins (SASPs), specifically β-SASP, α-SASP, and α/β-SASP, respectively, suggesting their potential as species-level biomarkers for *Bacillus* spores. Stump et al. (2005) subsequently confirmed *m*/*z* 6679 as a *B. anthracis* biomarker. Castanha and collaborators (2006), using MALDI-TOF MS, electrospray ionization ion-trap MS, and MS/MS, identified two SASP peaks present in all *B. anthracis*, *B. cereus*, and *B. thuringiensis* strains examined. The β-SASP peak varied in mass and sequence, appearing at 6679 *m*/*z* in *B. anthracis* and at 6695/6711 *m*/*z* in *B. cereus*/*B. thuringiensis*, while α-SASP consistently appeared at 6835 *m*/*z* in all strains.

In 2008, Callahan et al. identified *m*/*z* 6675 as specific to the *B. anthracis* Delta strain. In 2009, Lasch and collaborators identified *B. cereus* group biomarkers at *m*/*z* 5171, 5886, and 7368, whereas *B. anthracis* exhibited specific peaks at *m*/*z* 4606, 5413, and 6679. Nonetheless, none of these signals were entirely discriminative: the β-SASP signal at 6679 *m*/*z*—formerly considered species-specific—was also detected in some *B. cereus* spectra. Similarly, Dybwad et al. (2013) found the 6679 *m*/*z* peak in *B. cereus* DSM 8438, *B. cereus* R3, and *B. thuringiensis* BGSC 4CC1. The peak at *m*/*z* 5413, previously believed to be specific to *B. anthracis* spores, was detected even in the absence of spores, undermining its specificity.

As early as 2013, Jeong and collaborators identified several biomarkers specific to *B. anthracis* spores (*m*/*z* 2503, 3089, 3376, 6684, 6698, 6753, and 6840), among 30 peaks associated with the *Bacillus* species. Pauker et al. (2018) reported biomarkers at *m*/*z* 2445 and 4440 but did not detect those previously described by Lasch (2009), Dybwad (2013), or Castanha (2006). In 2021, Manzulli and colleagues identified species-specific signals by MALDI-TOF MS for *B. thuringiensis* (2956, 2968, and 3411 Da), *B. mycoides* (5422 Da), *B. anthracis* (3339, 3592, 4871, and 9740 Da), *B. weihenstephanensis* (4637, 7324, and 9272 Da), and *B. wiedmannii* (5443 Da). In 2020, Wei et al. reported *m*/*z* 3682.4, 5476.7, 6610.6, 6680.1, 7792.4, 9475.8, and 10934.1 as markers capable of differentiating *B. anthracis* from *B. cereus*.

Most recently, in 2025, a study from Argentina (Rocca et al.) identified nine biomarkers as specific to *B. anthracis*: 5413 Da, 6675–6679 Da, 2994 Da (highlighted as a primary biomarker via PCA), 4333, 5886, 7163, 9211, 15100, and 16000 Da. Although the peaks at 6675 and 6679 Da had previously been reported in *B. cereus*, the 2994 Da biomarker demonstrated strong statistical discrimination for *B. anthracis*.

Despite these advances, discriminating *B. anthracis* from its close relatives—*B. cereus* and *B. thuringiensis*—remains challenging. The search for truly species-specific biomarkers must therefore continue. Data on MALDI-TOF MS identification of *B. cereus* biovar *anthracis* (Bcbva) remain scarce. In 2014, the β-SASP marker once considered specific to *B. anthracis* was identified in CI and CA strains of Bcbva and in *B. cereus* B06020 using LC-MS/MS. Dupke and collaborators (2018) identified a protein uniquely found thus far in Bcbva strains—pXO2–60 (35 kDa) and confirmed its predicted cleavage site and N-terminal sequence by MALDI-TOF MS after protein extraction, further illustrating the absence of standardized methodologies for biomarker identification. In 2014, Chenau et al. emphasized that no relevant markers had yet been identified for *B. anthracis* spores, the primary form of environmental persistence and a central target of immunodetection assays.

Subsequent studies showed that in addition to the total number of biomarkers reported, the most critical factor is their precision across strain diversity, morphological states, and experimental conditions. Pauker et al. (2018) and Manzulli et al. (2021) analyzed a broad panel of *B. cereus* group strains and found that vegetative cell spectra generated by MALDI-TOF MS contain subsets of peaks that allow differentiation among *B. anthracis*, *B. cereus*, and other *B. cereus* group species. However, several peaks initially thought to be exclusive—particularly those in the 6.6–6.8 kDa range—proved to be shared when larger strain sets were examined.^75,81^

In 2023, Abdelli and collaborators demonstrated that the combined presence of subsets of these peaks in *B. anthracis*-like strains correlates with a greater genomic proximity to the anthrax clade.^85^ Thus, MALDI-TOF MS fingerprints reported in the literature not only distinguish species but also reveal proteomic and genomic clustering of anthracis-like strains toward *B. anthracis*, which is highly relevant for public health surveillance and the early detection of emerging lineages with anthrax-like pathogenic potential. Converging results from studies employing complementary approaches—including advanced statistical analyses and cross-validation with expanded strain panels—highlight that diagnostic reliability depends critically on well-defined mass-spectral panels.^7,46,72,73,86^

Within this context, techniques such as bottom-up LC-MS/MS have been employed to identify species-specific peptides of *B. anthracis* that complement and validate MALDI-TOF-defined biomarkers. In 2022, Witt and collaborators demonstrated that certain peptides derived from nuclear and surface proteins are conserved across *B. anthracis* strains and absent from closely related *B. cereus* group species, proposing a panel of peptide markers. Similarly, Bothra et al. (2023) used LC-MS to investigate the environmental regulation of toxin production (PA, LF, EF), linking toxin-associated biomarkers to the physiological state of the bacterium, a relevant consideration for interpreting results in animal models or real-world exposure scenarios.

Although numerous biomarkers have been identified over nearly three decades, many exhibit cross-reactivity with closely related species, particularly *B. cereus*, which compromises their specificity for biosurveillance and biodefense applications, as illustrated in Figure 1.

**Figure 1:**
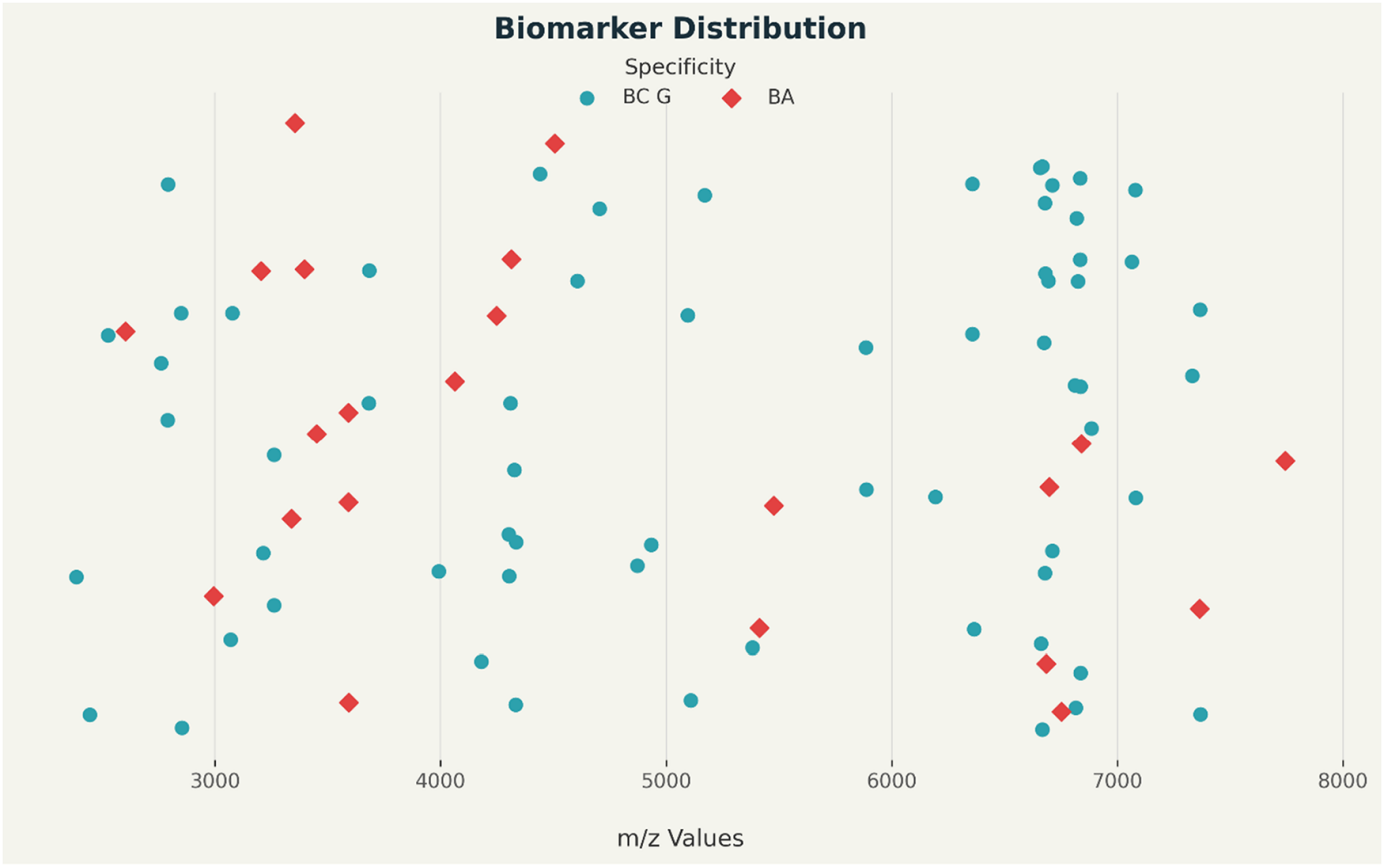
*Scatter plot of* *B. anthracis* *and* *B. cereus* *group biomarkers. The graph displays the distribution of 87 mass-spectral markers derived from* Table 2 *using Python. Points were plotted based on their m/z values (x-axis), with a randomized vertical dispersion. The legend distinguishes between BC G (markers present in the* *B. cereus* *group) and BA (markers specific to* *B. anthracis* *).* The scatter plot above demonstrates the specificity issues discussed in the literature. According to recent studies, the potential primary discriminants for *B. anthracis* are values *m*/*z*: 2603, 2994, 3203, 3339, 3353, 3396,3, 3450, 3590,4, 4063, 4248, 4313, 4505, 4606, 4871, 5413, 5476, 6610,6, 6684, 6753, 6840,7365,3, 7744.

### 4.2 Methodological Analysis

Methodological diversity among studies is a major reason for poor reproducibility and the lack of consensus on reference biomarkers for *B. anthracis*. Individual studies use different protocols for analytical assays and data analysis/interpretation, leading to equivocal results and precluding direct comparison across findings. Publications from 1996 to 2025 show 10 distinct categories with variations (Table 3).

**Table 3:** Differences among 17 Methods

methodological category	number of variations
Culture media	15 different
Laser shots	9 different ranges (50–2000)
Equipment	8 different models
Calibrators	7 different types
MALDI matrices	3 different types
Operating modes	6 different modes
Chemical/enzymatic extraction	6 different methods
Inactivation methods	4 different types
Cell disruption methods	4 different techniques
Laser type	2 different types

This underscores the need to focus on the identification, validation, and incorporation of truly species-specific biomarkers into reference databases, as well as on the implementation of analytical algorithms that explicitly prioritize specificity in order to ensure the accuracy and reliability of *Bacillus anthracis* identification using this technique. Nevertheless, several limitations of MALDI-TOF MS for *B. anthracis* discrimination have been repeatedly highlighted in the literature. These include inadequacies in current reference libraries, which do not sufficiently represent the genetic and phenotypic diversity of circulating strains, particularly those relevant to bioterrorism preparedness and atypical environmental isolates; a lack of methodological harmonization, resulting in variable and nonreproducible outcomes across laboratories; insufficient validation, since the specificity of numerous proposed biomarkers has not been systematically assessed against broad and diverse strain panels; and persistent quality-assurance shortcomings, exemplified by the absence of proficiency-testing programs and standardized performance metrics.^3^

In 2025, Lasch and colleagues developed a high-performance biosecurity (HPB) spectral library composed of thousands of MALDI-TOF MS spectra from BSL-3/4 pathogens, including *B. anthracis*. In this system, species-specific conserved “core” peaks are directly embedded into the main spectral profiles (MSPs), thereby enhancing interlaboratory reproducibility. In parallel, studies such as that by Florencia et al. (2025) explored the application of machine-learning approaches to MALDI-TOF MS-derived spectra for anthrax diagnosis and surveillance, enabling the extraction of the complete spectral fingerprint as a multidimensional feature vector in supervised models rather than relying on manual inspection of a limited set of *m*/*z* values.

When laboratories generate distinct biomarker profiles as a result of nonstandardized methodologies, comparison against a shared reference library becomes intrinsically challenging. Consequently, methodological standardization is required in the four core domains to overcome these limitations and enhance diagnostic robustness. Based on the analyses performed, a harmonized protocol was proposed encompassing the following domains (Table 4): sample-preparation standards: standardized growth conditions; uniform cell-lysis procedures; and use of equivalent sample quantities; analytical parameters: matrix composition; analytical mass range; instrument settings and calibration protocols; data-processing criteria (peak-detection thresholds, mass-tolerance parameters, minimum spectral-quality requirements, and matching algorithms with scoring systems that explicitly prioritize specificity); and quality-control requirements (daily performance checks, calibration verification, use of negative controls and sterile media, and routine participation in proficiency-testing programs).

**Table 4:** Suggested Protocol Based on Methods Described in the Supporting Material

step	description
Sample collection	Clinical or environmental sample collection
Culture and incubation	Plate on AK NR2 Agar or TSA; incubate at 37 °C for 18–24 h
Inactivation	Treat sample with 70% TFA or validated heat to ensure inactivation
Cell lysis	Mix 1 μL of sample suspension with 1 μL of matrix (HCCA + SA); pipet or vortex to homogenize
Spotting and drying	Spot mixture onto MALDI target plate and let air-dry
Instrument setup	HCCA 10 mg/mL in 50% acetonitrile/0.1% TFA; linear mode, 20 kV, daily calibration
Spectral acquisition	Collect ≥10 acceptable spectra per sample; include matrix and sterile medium as negative controls
Data processing	Baseline subtraction, Savitzky–Golay smoothing, S/*N* > 3, mass tolerance ±50 ppm
Automated matching/scoring	Match peaks to specific biomarker reference library; apply prioritization scoring
Quality control	Daily: analyze *B. anthracis* and *B. cereus* reference strains, calibrate instrument; quarterly proficiency testing
Result and genomics decision	If biomarkers are specific, report as positive; if ambiguous/failed, trigger PCR/genomic confirmation

## 5 Conclusion and Perspectives for the Future

The number of biomarkers reported across studies, together with the substantial variability observed, indicates a lack of robust reproducibility among experiments and among research groups. This, in turn, underscores the urgent need for methodological standardization, particularly with respect to the identification, validation, and incorporation of truly exclusive biomarkers into reference databases, as well as the implementation of analytical algorithms that explicitly prioritize specificity, to ensure the accuracy and reliability of *Bacillus anthracis* diagnosis using MALDI-TOF MS. Notably, several biomarkers proposed in the literature are not unique to *B. anthracis*, and some initially presumed to be species-specific have subsequently been detected in other *Bacillus* species. Furthermore, there remains a considerable gap in the MALDI-TOF MS characterization of *B. cereus* biovar *anthracis* (Bcbva), which is classified as a bioterrorism-relevant agent.

To advance diagnostic capabilities, future investigations employing optimized methodologies and innovative analytical strategies are essential. Promising avenues include machine-learning-based spectral pattern recognition; automated classification systems using weighted scoring and tiered biomarker hierarchies; genomic validation frameworks that integrate MALDI-TOF MS with rapid molecular assays; metabolomic complementation through LC-MS/MS; and the automation of sample-preparation workflows in high-biosafety laboratories. Collectively, these approaches represent the most promising path toward transforming the currently fragmented landscape of *B. anthracis* biomarkers into a coherent, reliable, and operationally robust set of tools for diagnosis, surveillance, and biodefense.

## Supplementary Material



## Notes

Comparative table summarizing key methodological parameters from MALDI-TOF MS studies on *Bacillus* species (1996–2025) (PDF)
