# Supplementary material for: Tracking Bacillus anthracis: The Legacy of MALDI-TOF Biomarkers in Scientific Literature, a Review
Source: ACS Omega. 2025 Dec 18;11(1):229–38. doi: 10.1021/acsomega.5c08472 (PMC12809309; doi:10.1021/acsomega.5c08472)
Supplement: Supplementary file 1 [file ao5c08472_si_001.pdf]

Tracking *Bacillus anthracis*: The Legacy of MALDI-TOF Biomarkers in Scientific Literature, a Review.

SUPPLEMENTARY MATERIAL

Supplementary Material 1: Summary of Key Methodological Parameters for *Bacillus* spp. Analysis by Mass Spectrometry

Method/ Procedure	Krishnamurthy, et al., 1996	Hathout, et al., 1999	Ryzhov, et al., 2000	Elhanany et al., 2001	Hathout, et al.,2003	Stump, et al, 2005	Castanha, et al.,2006	Callahan, et al., 2008	Lasch, et al., 2009	Dybwad, et al., 2013	Jeong, et al., 2013	Lasch, et al., 2015	Pauker, et al., 2018	Miyoung et al., 2019	Manzulli., et al., 2021	Wei, et al., 2020	Florencia, et al 2025
**Culture Medium**	TSA, Casein acid digestion, Leighton Doi, Blood Agar	NSM Sporulation	CDSM Sporulation	SSM Sporulation, Modified Schaeffer Broth, Nutrient Broth, MYP, USA, LB Agar, AK NR2 Agar	Casein acid digestio n, Nutrient Agar, NSM Sporulat ion, BS Schaeffe r without antibioti c	Blood Agar, Nutrient Agar	Blood Agar, Nutrient Agar	Blood Agar	Blood Agar	Modified Schaeffer broth, LB Agar	-	Nutrient Broth Sporulation	Blood Agar, Nutrien t Broth Sporul ation	MYP, USA	Blood Agar	-	Blood Agar
**Sample Preparation / Inactivation methods**	TFA, Gama Irradiation	TFA	TFA	TFA	TFA	TFA	TFA	TFA	TFA	Ethanol / Formic Acid	-	-	TFA	Ethanol / Formic Acid	TFA	Ethanol / Formic Acid; TFA	-
**Chemical/enzymatic extraction / Cell disruption methods**	Ultrasonication, Corona Plasma, Trypsin,	Corona Plasma	Ultra sonication, Corona Plasma, Lysozyme	CH3CN, TFA	N HCL_4	Trypsin, Pepsin, Formic Acid	-	Trypsin	-	-	-	-	-	-	-	-	-
**MALDI Matrix Type**	HCCA, SA	HCCA, SA, DHB	SA, DHB	HCCA, SA	SA	HCCA	HCCA	HCCA	HCCA	HCCA	HCCA	HCCA	HCCA	HCCA	HCCA	HCCA	HCCA
**Laser Shot**	100-200	50	50	50	50-100	100	100	600	2000	200	-	-	500	1000	500	500	240
**Sample Deposition Method**	Two-layers	Two-layers	Two-layers	Two-layers	Two-layers	Two-layers	Two-layers	Two-layers	Two-layers	Two-layers	Two-layers	Two-layers	Two-layers	Two-layers	Two-layers	Two-layers	-
**Chromatography**	-	-	-	-	HPLC	-	GC	-	-	-	-	-	-	-	-	-	-

**Method/ Procedure**	**Krishnamurthy, et al., 1996**	**Hathout, et al., 1999**	**Ryzhov, et al., 2000**	**Elhanany et al., 2001**	**Hathout, et al.,2003**	**Stump, et al, 2005**	**Castanha, et al.,2006**	**Callahan, et al., 2008**	**Lasch, et al., 2009**	**Dybwad, et al., 2013**	**Jeong, et al., 2013**	**Lasch, et al., 2015**	**Pauker, et al., 2018**	**Miyoung et al., 2019**	**Manzulli., et al., 2021**	**Wei, et al., 2020**	**Florencia, et al 2025**
**Calibrators**	Cytochrome C, Bovine Insulin	Linear	Linear, Bovine Insulin, Ubiquitin 1, Bovine Ubiquitin, Human Ubiquitin, Chicken Lysozyme, ACTH	Linear, Bovine Insulin Cytochrome C, Bovine Insulin, Trypsinogen, Angiotensin, Renin and ACTH	Linear, Bovine Insulin, Humana Ubiquitin	Linear	Linear	Bovine Insulin	Linear, Myoglobin, Peptideo Test Standard, In-house	Linear, Cytochrome C, Bovine Insulin, Ubiquitin 1	*E. coli*	*E. coli*	Linear	*E. coli*	Linear, *E. coli*	Linear, *E. coli*	- -
**Laser Type**	N2	N2	N2	N2	N2	N2	N2	N2	Nd:YA G	N2	N2	N2	Nd:YA G	N2	N2	N2	N2
**Equipment**	Vestec	Kratos	Kratos	Micromass ToF	Kratos	Bruker Ion trap	Bruker, Ultraflex	Bruker	Bruker. autoflex	Bruker, Autpflex	Bruker	Bruker	Bruker, Micro Flex LT	Bruker	Bruker	Bruker	Sirius Bruker

Legend 1: TSA = Tryptic Soy Agar; NSM = Nutrient Sporulation Medium; CDSM = Chemically Defined Sporulation Medium; SSM = Schaeffer Sporulation Medium; HCCA = α-cyano-4-hydroxycinnamic acid; SA = Sinapinic Acid; DHB = 2,5-dihydroxybenzoic acid; MYP = Mannitol Egg; HPTLC = High-Performance Liquid Chromatography; ACTH = Adrenocorticotropic hormone.
